# Evaluating the effectiveness of mindfulness-based interventions on rumination and negative emotions in Chinese University Students: A randomized controlled trial

**DOI:** 10.1371/journal.pone.0331084

**Published:** 2025-09-02

**Authors:** Keke Shi, Guoyan Feng, Meilin Ye, Xiaoyan Bi, Hongbo Cui, Weiyu Chen, Tao Gao, Zaihua Qing, Yankun Ma

**Affiliations:** 1 School of Education, Guangzhou University, Guangzhou, China; 2 Guangzhou Xinhua University, Guangzhou, China; 3 School of Public Administration, Guangzhou City Polytechnic, Guangzhou, China; 4 Mental Health Education and Counseling Center, Guangzhou University, Guangzhou, China; 5 Guangdong Industry Polytechnic University, Guangzhou, China; Alexandria University Faculty of Nursing, EGYPT

## Abstract

**Objectives:**

Rumination and negative emotions are prevalent among university students and are strongly linked to mental health disorders, including depression and anxiety. Group counseling involving a mindfulness-based strategies may help prevent university students from developing rumination and negative emotions and subsequent mental health disorders. This study aims to evaluate the alleviating effect of mindfulness intervention on rumination and negative emotions in university students in China.

**Methods:**

A randomized controlled trial (RCT) with 2 arms (Intervention Group and Control Group), three assessment time points (pre-intervention, post-intervention, and 3-month follow-up) is proposed. A total of 196 university students are randomly assigned to an intervention group (n = 98) receiving a 2-week, daily 1.5-hour mindfulness training (MT) and a control group (n = 98) receiving peer support (PS) sessions. Participants complete the Mindful Attention Awareness Scale (MAAS), Depression-Anxiety-Stress Scale (DASS-21), and Ruminative Responses Scale (RRS) before, immediately after, and three months post-intervention. Statistical analysis will compare outcomes between groups to evaluate the effectiveness using a repeated-measures ANOVA.

**Results:**

Before the intervention, no significant differences are observed between groups. After the intervention, the MT group shows significant improvements in MAAS scores and reductions in DASS-21 scores (p < 0.05) compared to the PS group. While immediate improvements in rumination (RRS) are not significant, the MT group exhibits significant reductions in rumination three months post-intervention.

**Conclusion:**

This study contributes to a better understanding of the effectiveness of mindfulness intervention in alleviating rumination and negative emotions in university students, and it is expected that with the proposed intervention university students can improve their psychological well-being. Besides, mindfulness interventions can potentially be extended to participants suffering from other psychological issues in the future.

## Introduction

Rumination refers to an individual’s tendency to focus their attention on negative emotions, repeatedly thinking about past negative feelings and experiences, and constantly pondering the causes and possible consequences of those negative experiences [1]. Altamirano et al. (2010) found a significant positive correlation between rigid thinking and rumination among university students in their study on cognitive mode [2]. Rumination can trigger negative emotions such as depression and anxiety [3,4]. Researchers in China also found a significant positive correlation between mental rigidity and rumination, and could significantly positively predict depression among university students [5]. Based on existing research results, psychological rigidity could promote the formation of rumination. Psychological rigidity refers to the thinking mode that individuals cannot flexibly adapt to changing situations. It is a typical characteristic of ruminant thinking that individuals with psychological rigidity have a lower ability to shift attention and often become entangled in negative experiences and events. The existing studies generally agreed that rumination was closely related to negative experiences, for example, it could cause social adaptation problems by hindering the positive thinking mode of individuals [1,6], significantly positively predict suicide ideas [7], depression level [7,8], stressful life events [7], insomnia [9], and alexithymia in university students [10]. These findings indicated that the key to improving the negative emotional experiences of individuals was to reduce rumination, and the key to alleviating rumination was to decrease the psychological rigidity of individuals. However, how to decrease the level of psychological rigidity? At present, there are few pieces of researches on intervention methods to alleviate rumination.

Mindfulness refers to being aware of current experiences with conscious attention and without judgment [11]. Being present is one of the core concepts of mindfulness, which emphasizes the ability to flexibly adjust attention and keep focusing on the feelings and experiences of the present moment. “Acceptance” and “cognitive disengagement” are the other two core concepts of mindfulness, which comprehensively reflect the openness of experiences, that is, to emphasize the acceptance of the original appearance of negative experiences, to coexist with negative thoughts and emotions without judgment, and to allow them to come and go freely in the mind at their own pace. Mindfulness cognitive therapy based on the concepts of focusing on the present, acceptance, and cognitive dissociation could effectively help individuals improve their mental flexibility [12,13]. Acceptance and cognitive separation are considered to be the main components of psychological flexibility. Previous studies found that psychological flexibility was significantly negatively correlated with negative emotions such as anxiety and depression [14]. Focusing on the present and psychological flexibility could significantly negatively predict the negative emotional problems of university students two months later [15]. The studies suggested that mindfulness intervention could improve the psychological flexibility of individuals, thereby improving the cognitive separation of individuals and the negative emotional experience. Besides, Acceptance and Commitment Therapy (ACT) holds that the root cause of psychological distress and disorders is psychological rigidity, and it emphasizes improving psychological rigidity and enhancing psychological flexibility through acceptance and change [16].

Existing researches have also shown that mindfulness training can effectively enhance the mental health of individuals at different age stages, including children and adolescents [17], college students [18,19], and the elderly [20]. A recent study has revealed how mindful parenting affects children’s emotional regulation abilities and its mechanism, providing strong support for the mindfulness intervention in the research [21].

As a non-invasive method based on psychotherapy, mindfulness intervention can help college students better manage emotional problems, reduce psychological pressure and improve their mental health. It has important reference value for the mental health intervention of college students in clinical practice. Mindfulness intervention is also a kind of psychological intervention method based on mindfulness training, which aims to improve mental health and quality of life by cultivating individuals’ awareness and acceptance of present experiences. The main mechanisms of mindfulness intervention include attention regulation, emotional regulation, cognitive defusion and enhanced mental flexibility. Besides, the application areas of mindfulness intervention include mental health, physical health, lifestyle improvement and so on. In other disciplines, especially psychology, mindfulness intervention has been used to great effect.

The remarkable characteristics of psychological rigidity were cognitive fusion and experiences avoidance [22], which were the opposite of the concepts of cognitive fusion and experiences avoidance in mindfulness. Greco et al. found that cognitive fusion and experiences avoidance could significantly negatively predict rumination [23]. Studies in China also found that mindfulness played a significant moderating role between mental rigidity and depression of university students; that is, the higher level of mindfulness, the less the impact of psychological rigidity on an individual’s depression level; on the contrary, the more significant the impact of psychological rigidity on depression [5]. Based on the previous analysis, this study suggests that the improvement of psychological rigidity is central to the reduction of rumination thinking. Furthermore, mindfulness intervention is considered an effective approach to reducing psychological rigidity, as it directly addresses this key factor that underpins both the enhancement of intervention outcomes and the alleviation of rumination thinking.

The primary objective of this study is to compare the efficacy of mindfulness intervention in alleviating rumination and negative emotions compared with the wait-list control group across three-time points (T1 = baseline assessment, T2 = immediately after the intervention, and T3 = three months after the intervention). Based on the above, this study proposes the hypothesis that mindfulness intervention can effectively alleviate rumination and negative emotions. More specifically, it is the psychological flexibility generated by the concept of focusing on the present, acceptance, and cognitive dissociation advocated by mindfulness, which can effectively improve psychological rigidity. The improvement of psychological rigidity will break the dreadful cycle of rumination, thus reducing rumination and improving the psychological health level of individuals. To verify this hypothesis, this study will test the effect of a mindfulness intervention on rumination and negative emotions of university students through a randomized control group experiment. The purpose of this study is to examine the effectiveness of the mindfulness intervention. Additionally, we are interested in how mindfulness enhanced psychological flexibility and reduced cognitive fusion, which are central to the theoretical framework of mindfulness-based interventions. This mechanism-focused approach is critical for advancing the theoretical understanding of mindfulness interventions and for refining their application in practice. Although there have been some studies on the improvement of mental flexibility and cognitive dissociation with mindfulness intervention, most of the existing studies have focused on the western cultural background, while Chinese college students are unique in terms of cultural background, educational environment and social pressure, which may affect the effect and mechanism of mindfulness intervention. Therefore, this study explores the applicability and effect of mindfulness intervention in this specific group, and provides empirical evidence for the promotion of mindfulness intervention in the Chinese context in the future. More importantly, this study is significant because its findings can help university administrators and teachers implement mindfulness interventions aimed at reducing rumination, negative emotions, and subsequent mental health disorders among their students.

## Materials and methods

### Ethics approval

The study was conducted in accordance with the Declaration of Helsinki, and approved by the ethics committee of the Institutional Review Board at Guangzhou Xinhua University (Document code: 2023L001) in July 14, 2023.

### Participants

**Fig 1:**
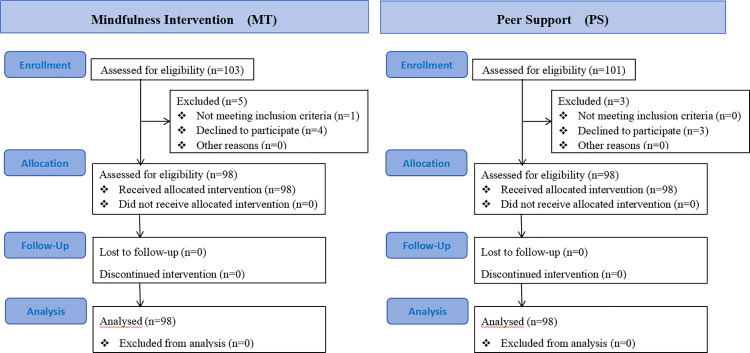
Shows CONSORT flow diagrams of the participants received mindfulness intervention and participants received peer support.

The participants were recruited online within the Guangzhou Xinhua university where the researchers worked, and the complete date range for participants recruitment and follow-up was from September 1, 2023 to January 30, 2024. A total of 204 participants signed up for the study. Due to various reasons, 8 dropped out during the intervention. A total of 196 participants completed the entire process and were randomly divided into a mindfulness intervention group (n = 98, with an average age of 20.05 ± 1.097, 30 males) and control group (n = 98, with an average age of 19.86 ± 0.908, 33 males) (Fig 1) The criteria for recruiting participants were university students who voluntarily wanted to participant should be recruited. Inclusion criteria were: Individuals with good cognitive abilities who were willing to participate were eligible. Exclusion criteria were: an insufficient cognitive state, in particular, presence of dementia or mild cognitive impairment. The participants were informed of the anonymous submission of the questionnaires, study’s purpose, and confidentiality agreement, and all the participants provided informed consent electronically before starting without any payment. Their privacy and anonymity were guaranteed. The participants could refuse or withdraw from the study anytime before submission. There were no important changes to methods after trial commencement (such as eligibility criteria).

A mixed model using treatment as a between-subjects factor (Group MT and PS) and time as a within-subjects factor (pre, post, and follow-up) will be tested. Interaction effect is powered. The R package sjstas [24], and pwr [25] are used for sample size calculation. The results indicate that a total of 157 participants will be required to detect a significant difference at an alpha level of 0.05 and a power of 80%. Accounting for potential dropouts, we conservatively estimate that 20% of participants may not complete the study. Hence, 196 participants are recruited (98 in each group).

### Study design

This study’s design was preregistered at the Chinese Clinical Trial Registry (ChiCTR2300067536). See [https://www.chictr.org.cn]. All assessments and procedures of this RCT were approved by the ethics committee of the Institutional Review Board at the Guangzhou Xinhua University. This study used a randomized controlled experimental design to compare the extent to which the control and intervention conditions affected the rumination and negative emotions of participants during pre-treatment, post-treatment, and three months after the treatment. All ongoing and related trials for this intervention were registered.

### Randomization and allocation concealment

In this study, permuted block randomization with an allocation ratio of 1:1 is carried out [26] to assign participants to either mindfulness training (MT) or wait-list control (PS). To implement this, we generate a computer-based random sequence and assign participants to groups based on odd and even positions within each block. Specifically, participants in odd positions are assigned to MT group, while those in even positions are assigned to PS group. This method ensures balanced group sizes and minimized selection bias. Blinded to the study assignment, participants are allotted to either group in a 1:1 ratio. Participants are randomly assigned to two groups: the intervention group receiving mindfulness intervention or the control group receiving standard treatment with non-therapeutic intervention.

### Blinding

Participants are unaware of the study’s randomization into the designated groups. Therefore, participants will not be informed of the group to which they are allocated.

Additionally, to minimize bias, the mindfulness intervention group and the control group will receive a 8-modules (with 2 weeks, once a day for 90 min each time). Participants in the control group carry out peer assistance intervention, mainly interactive communication, including eight activity themes: communication is an art, love to work hard to win and so on, which are carried out through video watching, live broadcast, sharing and discussion.

To maintain blinding throughout this study, the researchers will be blinded to participants’ randomized assignments into the groups. This process is carried out by a assistant who is not involved in this study. In addition, data collection for this study is conducted by the same research assistant, ensuring impartiality. The assistant will remain unknown to the study’s hypotheses. Moreover, data analysis for this study will be performed by statisticians who are not involved. The statistician will utilize a statistical analysis plan to conduct data analysis before the final unblinding of the data lock.

### Measurement variables

#### The Chinese version of mindful attention awareness scale.

The MAAS compiled by Brown and Ryan [27] and revised by Chen et al. [28] is adopted. Fifteen items are measured in the scale. The scale ranges from “almost always” (1 point) to “almost never” (6 points), and there are no reverse scoring questions. The average scores are calculated, with higher scores indicating higher levels of awareness and attention of the individual to the present. By using this scale, researchers may gain insights into the levels of mindful attention among different groups of people. In this study, the Cronbach’s α of the scale in this study is 0.890. This high value indicates that the items within the scale are highly consistent and measured the same construct effectively.

#### The depression-anxiety-stress scale.

The DASS compiled by Lovibond et al. [29] was modified by Wen et al. [30]. The scale has twenty-one items in total, including three sub scales of depression, anxiety, and stress, each with seven items, ensuring a balanced and thorough assessment of each emotional state. This scale allows researchers to not only obtain an overall measure of negative emotional states but also to examine the specific contributions of depression, anxiety, and stress individually, which is widely used across various populations to assess these emotional states. The Likert 4-point scoring method is adopted, ranging from “very inconsistent” (0 points) to “very consistent” (3 points). An average score for all items is calculated, with higher scores indicating higher levels of negative emotion in the participants. In this study, the Cronbach’s α of the scale in this study is 0.912, suggesting that the scale is robust and could be trusted to provide stable and accurate assessments of depression, anxiety, and stress in the study population.

#### The rumination response style scale.

The RRS compiled by Han and Yang [31]. The scale has twenty-two items, including three dimensions of symptomatic rumination, obsessive thinking, and introspection. Each dimension contains several items that collectively provides a comprehensive assessment of rumination across these different cognitive processes. The Likert 4-point scoring method is used, ranging from “almost never” (1 point) to “almost always” (4 points). An average score for all items is calculated, and higher average scores indicates higher levels of rumination, making it easy to interpret the results and compare them across different individuals or groups. The reliability of the RRS is a critical factor in ensuring the accuracy and consistency of the results. In this study, the Cronbach’s α of the scale is 0.903. This high value indicates that the items within the scale are highly consistent and reliably measure the intended constructs. The high reliability of the scale ensures that the results obtained are valid and can inform interventions and treatments aimed at reducing rumination and improving mental health.

### Quality control

In order to control the quality of the online questionnaire, the research group adheres to the principle of value neutrality and provides centralized training and guidance to the team members before the survey. Participants clarify the purpose of the study and agree that the general data collected will only be employed for the participant research. No guiding or suggestive language is used in the questionnaire filling process, and the analysis process is scientific to ensure the authenticity and reliability of the research data. The homologous bias is tested by Harman single-factor test.

### Intervention procedure and Intervention program

This research uses a randomized controlled experimental design for subject recruitment before randomly assigning them to the intervention group and control group. Potential participants will first be screened to determine their eligibility for study participation. Invitations to take part in this trial will follow. The investigator fully explains the objectives and methods of the study to participants before conducting the research and ensures that they sign Subject Information and Consent Form before enrolling in the research to ensure ethical considerations and participant access to the intervention. We will ensure the anonymity of participants and inform them of their right to withdraw from the research at any time, guaranteeing their autonomy and confidentiality. Before the start of the experiment (T1), the two groups of participants are pretested to test the homogeneity of the two groups of participants. At the end of the intervention (T2), the two groups of subjects are post-tested to test the improvement effect of a mindfulness intervention on rumination and negative emotions. Three months after the intervention (T3), the two groups of participants are followed up to test the continuity of the effects of a mindfulness intervention on rumination and negative emotions. The time arrangement, testing methods, and measuring tools of the three tests are consistent between the two groups.

Mindfulness-Based Cognitive Therapy (MBCT) created by Segal et al. [32] is the theoretical basis for Mindfulness intervention, and it is designed and adjusted according to the psychological characteristics of university students. The mindfulness intervention in this study includes eight modules: experiences avoidance vs. experiences acceptance, mindful attention, mindful awareness, focus on the present, automatic navigation mode vs. action mode, avoidance response, thoughts are just thoughts, and allowing everything to be as it is. Each module is equipped with corresponding mindfulness exercises, including mindful breathing, body scanning, mindful meditation, mindful eating raisins, mindful walking, mindful yoga, mindful sitting, mindful kindness, and other exercises. The intervention lasts about 2 weeks, once a day for 90 min each time. The mindfulness intervention group is operated and implemented by an instructor who has been engaged in mindfulness intervention for many years, and 4 university students are arranged as course assistants. Each mindfulness intervention course consists of four stages: (1) Review the themes and exercises of the previous course (15 min); (2) Explain the concept of mindfulness (30 min), which is divided into 14 different concept topics; (3) Mindfulness exercises (30 min), according to the theme throughout the course to conduct the corresponding mindfulness exercises; (4) Discuss and share (15 min), discuss doubts about the concept of mindfulness in class, and share feelings about mindfulness practice. See Table 1 for specific unit content and practice. The control group carries out peer assistance intervention, mainly interactive communication, including eight activity themes: communication is an art, love to work hard to win, finding small fortune, self-discipline to turn the world around, re-understanding yourself, happiness is so simple, travel happiness and fitness cannot forget, which are carried out through video watching, live broadcast, sharing and discussion. Peer assistance is implemented by another university teacher, and four university students are also arranged as teaching assistants. The time, duration, and frequency during the intervention course are the same as those of the mindfulness group. See Table 2 for specific unit content.

**Table 1:** Topic and content of mindfulness intervention course.

Unite	Unit Topic	Unit Content	Mindfulness Intervention
1	Mindfulness intervention introduction	Ice breaking; explain the concept of mindfulness; introduce the mindfulness class schedule plan	The difference between mindfulness practice and traditional mental intervention
2	Experience avoidance	Explain the characteristics and inertia of experience avoidance	Mindfulness breathing
3	Experience acceptance	Explain the ideas and implications of mindful acceptance	Three minutes breathing space
4	Mindful attention	Explain divergent attention, concentrated attention and open attention	Body scan
5	Focus on the present	Explain the idea and meaning of what as it is	Mindful sitting
6	automatic navigation mode	Explain the errors of automatic navigation mode	Two ways to know
7	Action mode	Explain the correct pattern of mindful thinking	Two ways to know
8	Avoidance response1	Explain the avoidance response	Be mindful of your emotions
9	Avoidance response2	Identify the characteristics of the avoidance response	Mindfulness ideas
10	Thoughts are just thoughts1	Thoughts are not Facts	Mindfulness ideas
11	Thoughts are just thoughts2	Separate your thoughts from your emotions	Be mindful of your emotions
12	Allowing everything to be as it is	Allow and let it be	Mindful eating raisins
13	Mindful awareness	Become aware of physical feelings, emotions, and thoughts using mindfulness patterns	Mindful Walking
14	Turn kindness into action	Actions affect emotions	Mindful kindness

**Table 2:** Topic and content of peer assistance course.

Unite	Unit Topic	Unit Content	Course Forms
1	Ice-breaking	The communication theme; the topics that the participants are interested in	We-Chat group chat
2	Become a family	Self-introduction and mutual understanding; Explain the purpose and process of the activity	Tencent video exchange
3	Communication is an art	Share communication barriers and ways to overcome them, and bombard with strengths and build confidence	Communication case sharing
4	Happiness is so simple	Share unforgettable happy experiences, interesting things, etc., seek happiness and embrace happiness	Watch funny videos
5	Love to work hard to win	Share the past of struggle and success; guide thinking about the future, and explore the dream	Watch motivational videos
6	Travel happiness	Share travel anecdotes with each other; guide the association and relax	Watch the travel v-log
7	Enjoy the pear garden culture	Share the views and experiences of traditional culture; enjoy the charm of culture, and cultivate sentiment	Watch the pear garden culture video
8	Spiritual healing	Share healing books, games, music, sports and find the beauty around you	Watch the healing videos
9	Fitness can not forget	Share sports experience, understand the relationship between sports and mentality, and explore the strategies of physical and mental relaxation	Watch sports and fitness videos
10	Self-discipline to turn the world around	Share the schedules of well-known self-disciplined bloggers; share the plan book and cultivate self-control	Watch a Video on self-discipline
11	Music treasures	Share playlists, listen to music together, and tell stories of music and healing	Live song request
12	Finding small fortune	Introduce “slow life” and “small true happiness” to discover the beauty of life together	Share small true happiness
13	Re-understanding yourself	Exchange with each other over the past two weeks of sentiment, contrast before and after the growth	Live discussion together
14	Be Thankful to have you	Summarize and share the 14-day harvest, and look forward to the future	Watch the memory recording video

In combination with the psychological characteristics of university students and the need for epidemic control and other actual situation, all the interventions in this study adopt the online teaching mode. The first course of the two groups of intervention mainly carries out ice-breaking and course introduction. The last course mainly includes a course review and explanation of precautions after the intervention, and the middle 12 courses are carried out successively according to the module theme.

### Statistical analysis

SPSS23.0 is used to conduct paired sample T-test and independent sample T-test for the experimental group and control group before, after, and three months after the intervention, and the intervention effect is tested by inter-group – intra-group and pre-intervention – post-intervention multidimensional test.

When baseline outcome values are reported, we calculate SMD using the ANCOVA estimate [33]. When missing from trial reports, within-study baseline–endpoint correlations are calculated from publicly available individual participant data (IPD) or are imputed as follows. For the other outcomes, we take the mean of the correlations available in other studies [34]. When baseline data are not available, we calculate SMD using adjusted (if available) or unadjusted final values analyses [35–37]. Missing standard deviations are imputed averaging those of other time points within the same study and outcome, or, if not available, from other studies using the same instrument. Sub scales are combined when possible using their correlations. Ordinal and categorical data are transformed to be pooled together with continuous data [34]. To estimate the magnitude of between-group differences at post-intervention and 3-month follow-up, effect sizes will be calculated by dividing the differences in means by the pooled standard deviation (SD).

## Results

### Comparison of variables between MT and PS before intervention

In order to test the homogeneity of the two groups of participants, an independent sample T-test is used to analyze the difference of pretest data of various variables in the mindfulness intervention group (MT) and peer group (PS). Table 3 shows no significant baseline differences between the MT and PS groups (p > 0.05), confirming the homogeneity of the two groups prior to intervention.

**Table 3:** The comparison of pretest variables between MT and PS (*M ± SD*).

Variable	MT	PS	*t*	*P*
MAAS Total	58.30 ± 12.27	59.47 ± 9.46	−0.75	0.454
RRS Total	46.02 ± 14.46	45.33 ± 10.87	0.38	0.704
RRS – Symptomatic rumination	24.02 ± 8.35	23.33 ± 6.53	0.65	0.518
RRS – Obsessive thinking	11.48 ± 3.73	11.26 ± 2.90	0.47	0.639
RRS – Introspection	10.52 ± 3.75	10.74 ± 2.63	−0.49	0.628
DASS Total	38.17 ± 11.88	38.10 ± 10.90	0.044	0.965
DASS – Stress	13.93 ± 4.67	13.28 ± 4.19	1.03	0.304
DASS – Anxiety	12.08 ± 3.88	11.88 ± 4.01	0.36	0.718
DASS – Depression	12.16 ± 4.21	12.05 ± 4.12	0.19	0.850

* MAAS indicates the level of mindful attention awareness, RRS indicates the level of rumination, and DASS indicates the level of stress-anxiety-depression.

### Difference test of variables before and after intervention between MT and PS

To test whether mindfulness intervention (MT group) can alleviate rumination and negative emotions compared to peer assistance (PS group), the independent sample T-test is used to analyze pre-test and post-test score differences. The results are shown in Table 4. In the mindful attention awareness test, the increment of the MT group is significantly higher than that of the PS group (t = 5.57, p < 0.001), indicating that the improvement of mindfulness level of the MT group is significantly higher than that of the control group after intervention. The increment of stress-anxiety-depression scale (t = −2.57, p < 0.05), stress sub scale (t = −2.98, p < 0.001), anxiety sub scale (t = −2.86, p < 0.001) and depression sub scale (t = −2.04, p < 0.05) in MT group is significantly higher than that in PS group. There is no significant difference between the two groups in the total scale and its sub scale of rumination (p > 0.05), indicating that mindfulness intervention has no significant advantage in relieving rumination.

**Table 4:** Difference test of variables before and after intervention between MT and PS.

Variable	Group	*n*	*M*	*SD*	*t*	*P*
MAAS Total	MT	98	12.30	2.21	5.57^***^	0.000
	PS	98				
RRS Total	MT	98	−1.13	2.52	−0.45	0.654
	PS	98				
RRS – Symptomatic rumination	MT	98	−1.20	1.45	−0.83	0.406
	PS	98				
RRS – Obsessive thinking	MT	98	−0.28	0.66	−0.83	0.677
	PS	98				
RRS – Introspection	MT	98	0.35	0.67	0.52	0.606
	PS	98				
DASS Total	MT	98	−5.20	2.03	−2.57^*^	0.011
	PS	98				
DASS – Stress	MT	98	−2.42	0.81	−2.98^***^	0.003
	PS	98				
DASS – Anxiety	MT	98	−2.12	0.74	−2.86^**^	0.005
	PS	98				
DASS – Depression	MT	98	−1.56	0.77	−2.04^*^	0.043
	PS	98				

*p<0.05; **p<0.01; ***p<0.001. **M**=Mean, **SD**=Standard Deviation.

### Comparison of different variables between MT and PS after intervention and three months after intervention

This study uses an independent samples t-test to compare the score differences (post-test score minus tracking score) of various variables between the mindfulness intervention group (MT) and peer assistance group (PS) immediately after intervention and three months later. As can be seen from Table 5. In the Mindful Attention Awareness test, the MT group’s improvement is significantly higher than the PS group’s (t = 2.96, p < 0.01), indicating that the mindfulness intervention has a sustained improvement effect on the mindfulness level of participants three months after the intervention. In the Rumination Responses test, the MT group shows significantly greater reductions in total rumination (t = −3.06, p < 0.01), symptom rumination (t = −2.83, p < 0.01), compulsive thinking (t = −2.78, p < 0.01), and introspection rumination (t = −2.83, p < 0.05) compared to the PS group, demonstrating that mindfulness intervention has a significant continuous improvement effect on rumination three months after the intervention. In the Depression-Anxiety-Stress test, the MT group has significantly greater reductions in total stress-anxiety-depression (t = −9.23, p < 0.001), stress (t = −5.92, p < 0.001), anxiety (t = −7.16, p < 0.001), and depression (t = −7.11, p < 0.001) compared to the PS group, indicating a sustained effect on improving negative emotions three months after the intervention. Overall, the mindfulness intervention has a significant sustained effects on mindfulness, rumination, and negative emotions, while peer assistance does not show such effects.

**Table 5:** Difference test of variables in MT and PS after post test and after 3 months intervention.

Variable	Group	*n*	*M*	*SD*	*t*	*P*
MAAS Total	MT	98	6.40	2.17	2.96^**^	0.004
	PS	98				
RRS Total	MT	98	−6.326	2.07	−3.06^**^	0.003
	PS	98				
RRS – Symptomatic rumination	MT	98	−3.19	1.13	−2.84^**^	0.005
	PS	98				
RRS – Obsessive thinking	MT	98	−1.60	0.58	−2.78^**^	0.006
	PS	98				
RRS – Introspection	MT	98	−1.52	0.67	−2.84^*^	0.024
	PS	98				
DASS Total	MT	98	−10.24	1.11	−9.23^***^	.000
	PS	98				
DASS – Stress	MT	98	−3.78	0.64	−5.92^***^	.000
	PS	98				
DASS – Anxiety	MT	98	−3.33	0.47	−7.16^***^	.000
	PS	98				
DASS – Depression	MT	98	−3.33	0.47	−7.11^***^	.000
	PS	98				

### Comparison between MT and PS before intervention, after, and three months after intervention

**Fig 2:**
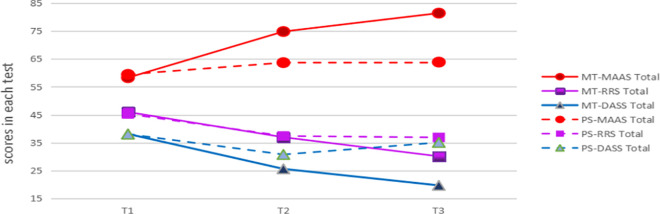
Shows the variation trends in mindfulness (MAAS), rumination (RRS), and negative emotions (DASS-21) for the MT and PS groups at baseline, post-intervention, and three-month follow-up.

The variation trend chart of each variable is made for the test scores of the two groups before, after, and three months after intervention (Fig 2). According to Fig 2, there are no significant differences in the total score of mindful attention awareness, rumination and negative emotion between the two groups before intervention (T1), and the two groups are homogeneous. After the intervention (T2), scores of mindful attention awareness of the two groups of participants are significantly improved, and the MT group increases more. The total scores of stress-anxiety-depression and rumination of the two groups are decreased, and the MT group decreases more, indicating that the mindfulness intervention is better than peer assistance in improving the level of mindfulness awareness of university students and reducing negative emotions and rumination. Three months after the intervention (T3), scores of mindful attention awareness in the MT group continues to improve and are significantly higher than that in the PS group, while the level of mindful awareness in the PS group has almost no change, which indicates that mindfulness practice can continuously improve the level of mindful attention awareness of university students. The total scores of stress-anxiety-depression in the MT group continue to decrease and are significantly lower than that of the PS group, while the total scores of stress-anxiety-depression in the PS group increases significantly, indicating that mindfulness practice can continue to relieve negative emotions, while peer assistance lacks the continuity of the effect of alleviating negative emotions. The total scores of rumination in the MT group continue to decrease and are significantly lower than that in the PS group, while the scores of rumination in the PS group are almost unchanged, indicating that mindfulness practice continues to reduce participants’ rumination, while peer assistance has no sustained effect on the improvement of rumination.

## Discussion

Before the intervention, the two groups are homogeneous in negative emotional states (including stress, anxiety, and depression) and rumination (including symptomatic rumination, obsessive thinking, and introspective rumination). After the intervention, the negative emotional states and rumination of the two groups shows significantly relief, but the improvement effect of the mindfulness intervention group is more prominent. Three months after the intervention, the mindfulness intervention continues to have a significant sustained effect on the participants’ improvement of rumination and negative emotions, while the effect of peer assistance on the improvement of rumination and negative emotions has disappeared.

### In terms of negative emotions improvement

Previous studies have found that mindfulness intervention can effectively reduce the negative emotional experiences of individuals [38,39], which are consistent with the results of this study. This study shows that the significant reduction in negative emotions in the experimental group is due to the concept of “experience openness” formed by the comprehensive effects of “acceptance” and “focus on the present” in mindfulness intervention. Paticipants in the experimental group gradually understand the connotation of the concept of “acceptance” through mindfulness intervention and master the method of coexisting with negative emotions. Specifically, they learn to accept the original appearance of negative experiences, coexist with negative thoughts and emotions without judgment, and allow these thoughts and emotions to come and go freely in the minds at their own pace, thus saving the psychological resources consumed by habitual resistance to negative emotions in the past. By reallocating these limited psychological resources to the tasks at hand, participants achieve “focus on the present”. Through the comprehensive effect of “accepting” and “focusing on the present”, the participants learn “experiential openness”; that is, they no longer obsess about eliminating, reducing, or fighting negative emotions, but invest the psychological resources saved by “acceptance” of negative emotions into “focus on the present” with an open mind. After successfully shifting their attention, they reduce their attention and perception of negative emotions. This shift is key to effectively alleviating negative emotions of the participants in the mindfulness intervention group.

The negative emotions of the control group are relieved due to interpersonal communication and interaction in peer intervention. Previous studies have found that peer assistance has a certain effect on relieving negative emotions [40,41]. However, the intervention effect of peer assistance is not sustained. In this study, three months after the end of the intervention, the negative emotions of the mindfulness group are significantly lower than those at the end of the intervention, while the negative emotion scores of the peer group are significantly increased. This result indicates that the improvement effect of peer assistance on negative emotions is likely to be temporary; that is, the intervention effect is difficult to maintain. Why is the continuation of the peer assistance intervention group poor? This study suggests that peer assistance only transfers the participants’ attention through interpersonal interaction. Once this external force disappears, the participants still lack the ability and methods to self-adjust and cope with negative emotions, and are easily trapped in the entanglement and confrontation with negative emotions. As a result, their perception of negative emotions is constantly emphasized, leading to the failure to continuously reflect the intervention effect. This also indirectly confirms that the improvement of negative emotions by mindfulness intervention is due to the change of the thinking mode of the participants in getting along with negative emotions, and participants have mastered the skills to coexist with negative emotions, so the continuous improvement effect of mindfulness intervention is longer.

The continuous improvement effect of a mindfulness intervention on negative emotions has also been confirmed in previous studies. Hong et al. (2020) found that the improvement effect of the mindfulness intervention on test anxiety in university students lasted for two months after the intervention [42]. Fan et al. found that the improvement effect of a mindfulness intervention on anxiety and depression could last three months after the intervention [43]. However, some studies found that the effect of mindfulness intervention had no obvious continuity. For example, Chen et al. (2017) found that the original effect of relieving anxiety, depression, and compulsion disappeared six months after the end of a mindfulness intervention [44], and the reason was that the participants did not continue to practice mindfulness after mindfulness intervention ended. This study suggests that long-term mindfulness practice is necessary to regulate negative emotions. Therefore, after the intervention, the research team monitors and requires participants in the experimental group to continue mindfulness exercises for at least 10 minutes daily via We-Chat small program clock-in, which is key to the good continuation effect of mindfulness intervention in this study. Mindfulness intervention is a gradual process. As mindfulness practice continues, individuals can further absorb and internalize the concepts of “acceptance” and “focus on the present”. This is key to mindfulness intervention effectively and continuously helping individuals relieve negative emotions.

### In terms of rumination improvement

Previous studies have indicated that mindfulness intervention may effectively promote the development of cognition [45,46] and play a role in deautomatization [47]. This study suggests that the significant improvement of rumination in the experimental group can be attributed to the concept of “psychological flexibility” formed by the comprehensive effect of “acceptance” and “cognitive dissociation” of mindfulness intervention. Participants in the experimental group gradually learn to accept negative emotions and negative thoughts through mindfulness intervention. To accept negative emotions is to coexist with negative emotions as mentioned earlier. To accept negative thoughts is to recognize the idea that “thoughts are just thoughts, not facts”, and to coexist with thoughts in an empirically open way. On the basis of “acceptance”, participants can transition to the process of separating “thoughts”, especially negative thoughts, from “facts” to achieve “cognitive dissociation”. That is, they are no longer entangled in negative thoughts and unable to extricate themselves, and no longer entangled in various negative emotions due to the entanglement of thoughts. In this approach, individuals can achieve “psychological flexibility” and break the vicious circle brought about by the “psychological rigidity” mode, thus alleviating rumination. Relevant research have also found that the positive intervention effect is attributed to the fact that mindfulness intervention may help individuals break rigid thinking, enhance cognitive flexibility, and significantly improve their emotional regulation ability [48,49].

The improvement in rumination in the control group is only temporary and benefits from the reduction in negative emotions. Negative emotions are the prerequisite for rumination. After peer assistance intervention, participants’ negative emotions are reduced, and so is rumination. However, similar to the situation of negative emotions, the improvement of rumination in the control group is only temporary, and the effect does not continue. This study indicates that three months after the intervention, the level of rumination in the mindfulness group is significantly lower than that at the end of the intervention and is significantly lower than that of the peer assistance control group. In contrast, the level of rumination in the peer assistance control group does not change significantly. This data result confirms that the “psychological flexibility” formed by mindfulness intervention is the fundamental factor for the improvement of rumination. In contrast, peer assistance only temporarily alleviates rumination, which is more likely related to the disappearance of external stress events, such as the regression of COVID-19 epidemic risk. The intervention in this study started during the period of closed management of the epidemic situation in a university and ended with the release of the epidemic situation. Therefore, the disappearance of the crisis factors caused by the epidemic situation might be an important external factor influencing the reduction of negative emotions and rumination. Previous studies found that under the stimulation of COVID-19, individuals experienced different levels of psychological stress reactions, such as anxiety, depression, sleep disorders, interpersonal sensitivity, and fear of infection [38,50]. University students, in particular, were vulnerable to psychological stress reactions under public stress events. When the stimulus of COVID-19 disappeared, the anxiety and other psychological stress reactions of the participants also subsided, and the rumination would also decrease with the significant reduction of negative emotions. Therefore, compared with the immediate effect of the intervention, the continuous effect of the intervention is more persuasive, and the advantages of mindfulness intervention in alleviating rumination are also highlighted.

To sum up, the advantages of mindfulness intervention in alleviating negative emotions are highlighted at the end of the intervention, while the advantages of the improvement effect of rumination are not highlighted until three months after the intervention. This study indicates that the reason is that the transformation of rumination involves a deep cognitive level. It requires individuals to frequently internalize the idea of mindfulness and adhere to its practice over a long time, gradually transforming their thinking mode at the underlying logical level. This process transforms the original “psychological rigidity” cognitive mode into a “psychological flexibility” cognitive mode, and breaks rumination from the root of thinking, thereby alleviating rumination. In addition, this study suggests that the reason why mindfulness intervention is superior to peer assistance in the continuation of negative emotions and rumination is that mindfulness intervention changed the individual’s thinking mode from the cognitive behavior level, while peer assistance only transfers the individual’s attention from the external levels of companionship and interaction. Therefore, the latter can only play a temporary and superficial improvement effect. The contributions of this study not only provide new insights for the field of psychology, but also offer reference for other relevant disciplines (such as education, public health, etc.) when designing intervention programs.

## Conclusions

In conclusion, Mindfulness-based interventions offer a promising, sustainable approach to reducing rumination and negative emotions in university students. This study contributes to a better understanding of the effectiveness of mindfulness intervention in alleviating rumination and negative emotions in university students. It is expected that with the proposed intervention university students can improve their psychological well-being. Incorporating mindfulness into educational programs may significantly enhance student well-being and mental health resilience. Additionally, mindfulness interventions can potentially be extended to participants suffering from other psychological issues in the future.

### Limitations and future research directions

There are still some limitations in this study. First, this study is limited by its focus on a single region in China so it is better to mention that future research should include diverse cultural contexts and longitudinal follow-ups to assess the sustainability of mindfulness interventions beyond three months. Second, the study design relies on self-report measures, which can introduce response biases. In order to solve this issue, participants should be assured of the confidentiality and anonymity of their responses to encourage honest reporting. Third, this study is only conducted on college students from China. Future researches should be conducted cross-culturally to compare the differences between Chinese and other countries’ college students between different groups to expand the general applicability of the findings. Besides, the online intervention format used in this study may have introduced certain biases. While it provides convenience and accessibility, it is also susceptible to factors such as network instability, variations in technical equipment, and individual students’ ability to adapt to online learning. Although measures are taken during the research process to mitigate these potential biases, including providing technical training to all participants prior to the intervention and establishing a technical support hotline, these steps may not have entirely eliminated all potential issues. Despite these limitations, further exploration is needed to identify the optimal duration of mindfulness practice for sustained psychological benefits. Additionally, integrating mindfulness with existing university counseling services can enhance its accessibility and impact.

## Supporting information

S1 Dataset Dataset used for analyses in present study. (SAV)

S2 Checklist CONSORT-2010-Checklist. (DOC)

S3 File The name of the registry and the registration number. (DOCX)

S4 File Completed CONSORT flowchart. (DOCX)

S5 Protocol Study protocol. (DOCX)
