# Supplementary material for: Evaluating the effectiveness of mindfulness-based interventions on rumination and negative emotions in Chinese University Students: A randomized controlled trial
Source: PLoS One. 2025 Sep 2;20(9):e0331084. doi: 10.1371/journal.pone.0331084 (PMC12404387; doi:10.1371/journal.pone.0331084)
Supplement: S3 File — (DOCX) [file pone.0331084.s003.docx]

This study’s design was preregistered at the Chinese Clinical Trial Registry (ChiCTR2300067536), see [https://www.chictr.org.cn]. All assessments and procedures of this RCT were approved by the ethics committee of the Institutional Review Board at the Guangzhou Xinhua University.
