# Supplementary material for: Evaluating the effectiveness of mindfulness-based interventions on rumination and negative emotions in Chinese University Students: A randomized controlled trial
Source: PLoS One. 2025 Sep 2;20(9):e0331084. doi: 10.1371/journal.pone.0331084 (PMC12404387; doi:10.1371/journal.pone.0331084)
Supplement: S4 File — (DOCX) [file pone.0331084.s004.docx]

**Mindfulness Intervention (MT)**

**()**

**Peer Support (PS)**

**Enrollment**

Assessed for eligibility (n=103)

Assessed for eligibility (n=98)

- Received allocated intervention (n=98)
- Did not receive allocated intervention (n=0)

Excluded (n=5)

- Not meeting inclusion criteria (n=1)
- Declined to participate (n=4)
- Other reasons (n=0)

**Allocation**

**Follow-Up**

Lost to follow-up (n=0)

Discontinued intervention (n=0)

**Analysis**

Analysed (n=98)

- Excluded from analysis (n=0)

**Enrollment**

Assessed for eligibility (n=101)

Assessed for eligibility (n=98)

- Received allocated intervention (n=98)
- Did not receive allocated intervention (n=0)

Excluded (n=3)

- Not meeting inclusion criteria (n=0)
- Declined to participate (n=3)
- Other reasons (n=0)

**Allocation**

**Follow-Up**

Lost to follow-up (n=0)

Discontinued intervention (n=0)

**Analysis**

Analysed (n=98)

- Excluded from analysis (n=0)
