# Supplementary material for: Evaluating the effectiveness of mindfulness-based interventions on rumination and negative emotions in Chinese University Students: A randomized controlled trial
Source: PLoS One. 2025 Sep 2;20(9):e0331084. doi: 10.1371/journal.pone.0331084 (PMC12404387; doi:10.1371/journal.pone.0331084)
Supplement: S5 Protocol — (DOCX) [file pone.0331084.s005.docx]

**Study Protocol**

**Study design**

This study’s design was preregistered at the Chinese Clinical Trial Registry (ChiCTR2300067536), see [https://www.chictr.org.cn]. All assessments and procedures of this RCT were approved by the ethics committee of the Institutional Review Board at the Guangzhou Xinhua University. This study used a randomized controlled experimental design to compare the extent to which the control and intervention conditions affected the rumination and negative emotions of participants during pretreatment, posttreatment, and three months after the treatment. All ongoing and related trials for this intervention are registered.


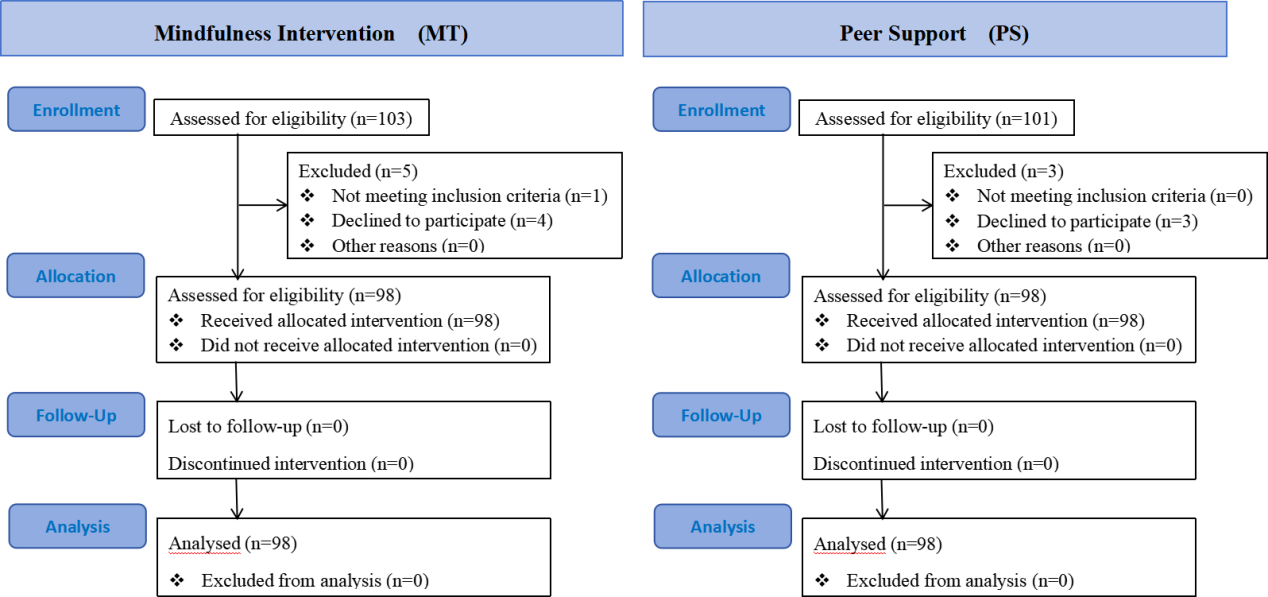


Fig.1 Completed CONSORT diagrams

**Quality control**

In order to control the quality of the online questionnaire, the research group adhered to the principle of value neutrality and provided centralized training and guidance to the team members before the survey. Participants clarified the purpose of the study and agreed that the general data collected would only be employed for the participant research. No guiding or suggestive language was used in the questionnaire filling process, and the analysis process was scientific to ensure the authenticity and reliability of the research data. The homologous bias was tested by Harman single-factor test.

**Intervention program**

Mindfulness-Based Cognitive Therapy (MBCT) created by Segal et al. (22) was the theoretical basis for Mindfulness intervention, and it was designed and adjusted according to the psychological characteristics of university students. The mindfulness intervention in this study included eight modules: experiences avoidance vs. experiences acceptance, mindful attention, mindful awareness, focus on the present, automatic navigation mode vs. action mode, avoidance response, thoughts are just thoughts, and allowing everything to be as it is. Each module was equipped with corresponding mindfulness exercises, including mindful breathing, body scanning, mindful meditation, mindful eating raisins, mindful walking, mindful yoga, mindful sitting, mindful kindness, and other exercises. The intervention lasted about 2 weeks, once a day for 90min each time. The mindfulness intervention group was operated and implemented by an instructor who had been engaged in mindfulness intervention for many years, and 4 university students were arranged as course assistants. Each mindfulness intervention course consisted of four stages: (1) Review the themes and exercises of the previous course (15min);  (2) Explain the concept of mindfulness (30min), which was divided into 14 different concept topics; (3) Mindfulness exercises (30min), according to the theme throughout the course to conduct the corresponding mindfulness exercises;  (4) Discuss and share (15min), discuss doubts about the concept of mindfulness in class, and share feelings about mindfulness practice. See Table 1. for specific unit content and practice. The control group carried out peer assistance intervention, mainly interactive communication, including eight activity themes: communication is an art, love to work hard to win, finding small fortune, self-discipline to turn the world around, re-understanding yourself, happiness is so simple, travel happiness and fitness cannot forget, which are carried out through video watching, live broadcast, sharing and discussion. Peer assistance was implemented by another university teacher, and four university students were also arranged as teaching assistants. The time, duration, and frequency during the intervention course were the same as those of the mindfulness group. See Table 2. for specific unit content.

In combination with the psychological characteristics of university students and the need for epidemic control and other actual situation, all the interventions in this study adopted the online teaching mode. The first course of the two groups of intervention mainly carried out ice-breaking and course introduction. The last course mainly included a course review and explanation of precautions after the intervention, and the middle 12 courses were carried out successively according to the module theme.

**Intervention procedure**

Before the start of the experiment (T1), the two groups of participants were pretested to test the homogeneity of the two groups of participants. At the end of the intervention (T2), the two groups of subjects were post-tested to test the improvement effect of a mindfulness intervention on rumination and negative emotions. Three months after the intervention (T3), the two groups of participants were followed up to test the continuity of the effects of a mindfulness intervention on rumination and negative emotions. The time arrangement, testing methods, and measuring tools of the three tests were consistent between the two groups.

**Statistical analysis**

SPSS23.0 was used to conduct paired sample T-test and independent sample T-test for the experimental group and control group before, after, and three months after the intervention, and the intervention effect was tested by inter-group - intra-group and pre-intervention - post-intervention multidimensional test.

| **Table 1.** Topic and content of mindfulness intervention course | | | |
| --- | --- | --- | --- |
| **Unite** | **Unit Topic** | **Unit Content** | **Mindfulness intervention** |
| 1 | Mindfulness intervention introduction | Ice breaking; explain the concept of mindfulness; introduce the mindfulness class schedule plan | The difference between mindfulness practice and traditional mental intervention |
| 2 | Experience avoidance | Explain the characteristics and inertia of experience avoidance | Mindfulness breathing |
| 3 | Experience acceptance | Explain the ideas and implications of mindful acceptance | Three minutes breathing space |
| 4 | Mindful attention | Explain divergent attention, concentrated attention and open attention | Body scan |
| 5 | Focus on the present | Explain the idea and meaning of what as it is | Mindful sitting |
| 6 | automatic navigation mode | Explain the errors of automatic navigation mode | Two ways to know |
| 7 | Action mode | Explain the correct pattern of mindful thinking | Two ways to know |
| 8 | Avoidance response1 | Explain the avoidance response | Be mindful of your emotions |
| 9 | Avoidance response2 | Identify the characteristics of the avoidance response | Mindfulness ideas |
| 10 | Thoughts are just thoughts1 | Thoughts are not Facts | Mindfulness ideas |
| 11 | Thoughts are just thoughts2 | Separate your thoughts from your emotions | Be mindful of your emotions |
| 12 | Allowing everything to be as it is | Allow and let it be | Mindful eating raisins |
| 13 | Mindful awareness | Become aware of physical feelings, emotions, and thoughts using mindfulness patterns | Mindful Walking |
| 14 | Turn kindness into action | Actions affect emotions | Mindful kindness |
| **Table 2.** Topic and content of peer assistance course | | | |
| **Unite** | **Unit Topic** | **Unit Content** | **Course Forms** |
| 1 | Ice-breaking | The communication theme：the topics that  the participants are interested in | We-Chat group chat |
| 2 | Become a family | Self-introduction and mutual understanding; Explain the purpose and process of the activity | Tencent video exchange |
| 3 | Communication is an art | Share communication barriers and ways to overcome them, and bombard with strengths and build confidence | Communication case sharing |
| 4 | Happiness is so simple | Share unforgettable happy experiences, interesting things, etc., seek happiness and embrace happiness | Watch funny videos |
| 5 | Love to work hard to win | Share the past of struggle and success; guide thinking about the future, and explore the dream | Watch motivational videos |
| 6 | Travel happiness | Share travel anecdotes with each other; guide the association and relax | Watch the travel v-log |
| 7 | Enjoy the pear garden culture | Share the views and experiences of traditional culture; enjoy the charm of culture, and cultivate sentiment | Watch the pear garden culture video |
| 8 | Spiritual healing | Share healing books, games, music, sports and find the beauty around you | Watch the healing videos |
| 9 | Fitness can not forget | Share sports experience, understand the relationship between sports and mentality, and explore the strategies of physical and mental relaxation | Watch sports and fitness videos |
| 10 | Self-discipline to turn the world around | Share the schedules of well-known self-disciplined bloggers; share the plan book and cultivate self-control | Watch a Video on self-discipline |
| 11 | Music treasures | Share playlists, listen to music together, and tell stories of music and healing | Live song request |
| 12 | Finding small fortune | Introduce "slow life" and "small true happiness" to discover the beauty of life together | Share small true happiness |
| 13 | Re-understanding yourself | Exchange with each other over the past two weeks of sentiment, contrast before and after the growth | Live discussion together |
| 14 | Be Thankful to have you | Summarize and share the 14-day harvest, and look forward to the future | Watch the memory recording video |
